# Supplementary figures and images for: Widespread prevalence of a methylation-dependent switch to activate an essential DNA damage response in bacteria
Source: PLoS Biol. 2024 Mar 11;22(3):e3002540. doi: 10.1371/journal.pbio.3002540 (PMC10957082; doi:10.1371/journal.pbio.3002540)

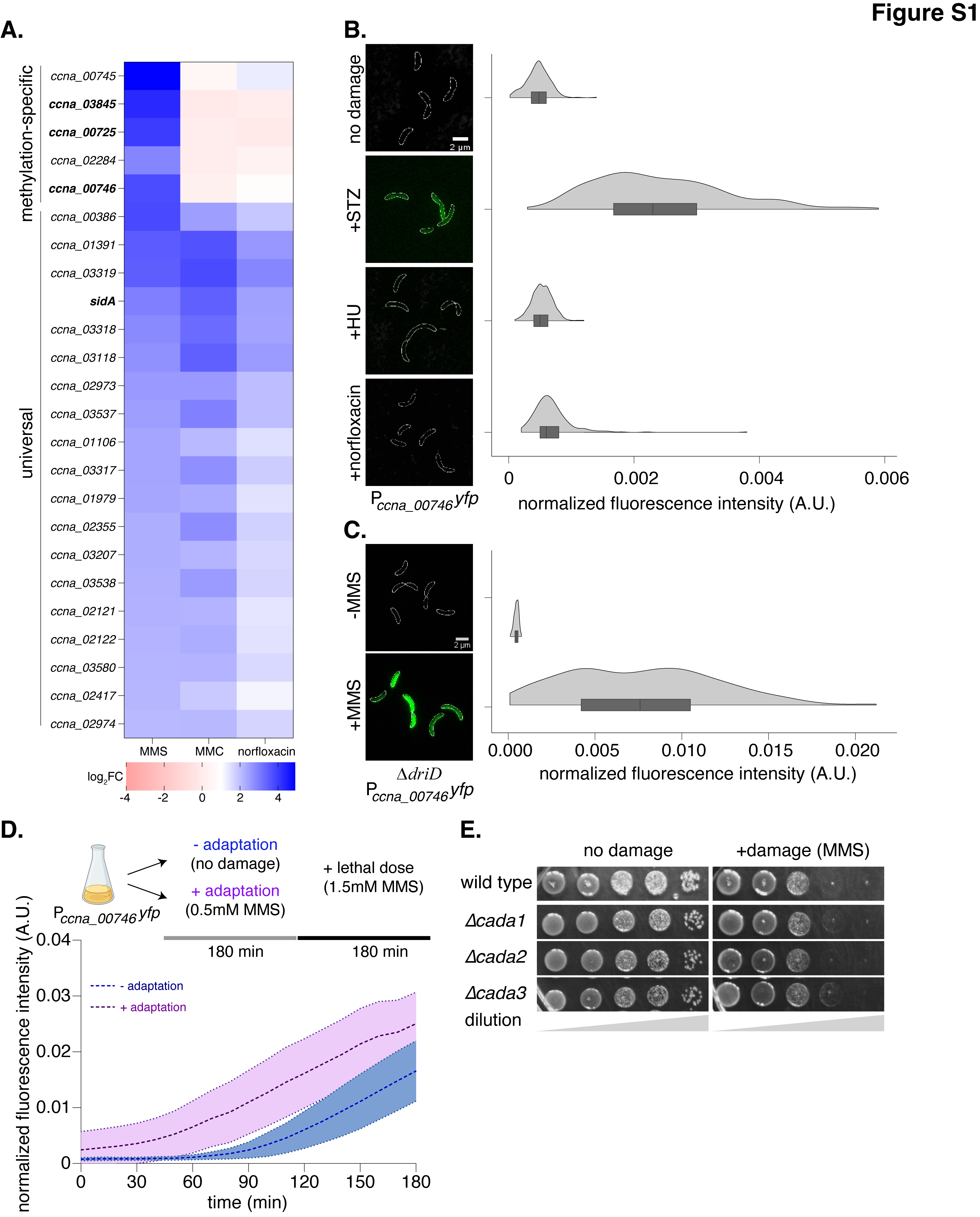

Supplement: S1 Fig — (A) Heat map of log2FC values for genes up-regulated in wild-type cells following MMS, MMC, or Norfloxacin treatment. (B) [Left] Representative cells showing Pccna_00746-yfp reporter induction upon exposure to STZ, MMC, norfloxacin, and HU. [Right] Violin plots show fluorescence intensity distribution normalized to cell area from single cells (n = 300, from 3 biological replicates). The underlying data are available in S1 Data. (C) [Left] Representative cells showing Pccna_00746-yfp reporter induction in ΔdriD background under 1.5 mM MMS damage. [Right] Violin plots showing fluorescence intensity distribution normalized to cell area from single cells (n = 300, from 3 biological replicates). The underlying data are available in S1 Data. (D) [Top] Schematic of the experimental protocol for testing the adaptive property of the Caulobacter methylation-specific damage response. Cultures of Pccna_00746-yfp cells were exposed to a sublethal dose of 0.5 mM either MMS (adapted) or no MMS (non-adapted). The cells were subsequently exposed to a higher dose of 1.5 mM MMS on agarose pads supplemented with PYE medium. [Bottom] Normalized fluorescence intensity kinetics was measured via time lapse microscopy over 3 h of lethal MMS exposure. Dotted lines (in dark) indicate mean time trace of induction kinetics while the shaded region (in light) indicates the standard deviation of all time traces for the respective conditions (here and for all other time lapse data) (n = 25). The underlying data are available in S1 Data. (E) Survival assay of individual deletions of cada genes with and without MMS exposure (1.5 mM). (TIF) [file pbio.3002540.s001.tif]

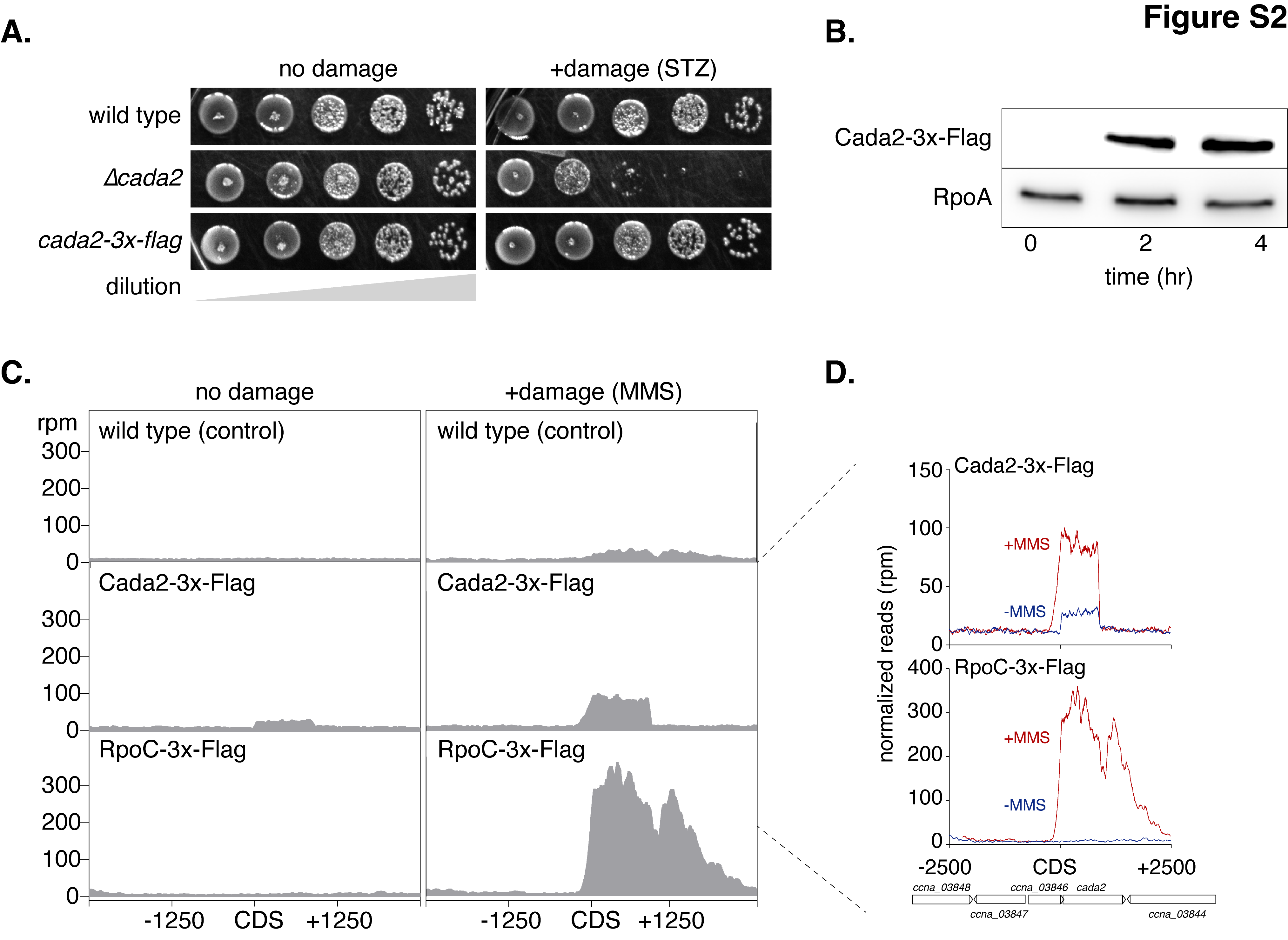

Supplement: S2 Fig — (A) Survival assay of cada2-3x-flag strain in the presence or absence of STZ damage (5 μg/ml). (B) Western blot showing Cada2-3x-flag levels at 0, 2, and 4 h after 1.5 mM MMS exposure. As a loading control, RpoA is probed. (C) ChIP-seq profiles for wild type (control), Cada2-3x-Flag, or RpoC-3x-Flag ±2.5 kb around cada2 CDS before (no damage) and after (+damage) exposure to 1.5 mM MMS. (D) Zoomed-in ChIP-seq profiles for Cada2-3x-Flag and RpoC-3x-Flag ±2.5 kb around cada2 CDS before and after exposure to 1.5 mM MMS. (TIF) [file pbio.3002540.s002.tif]

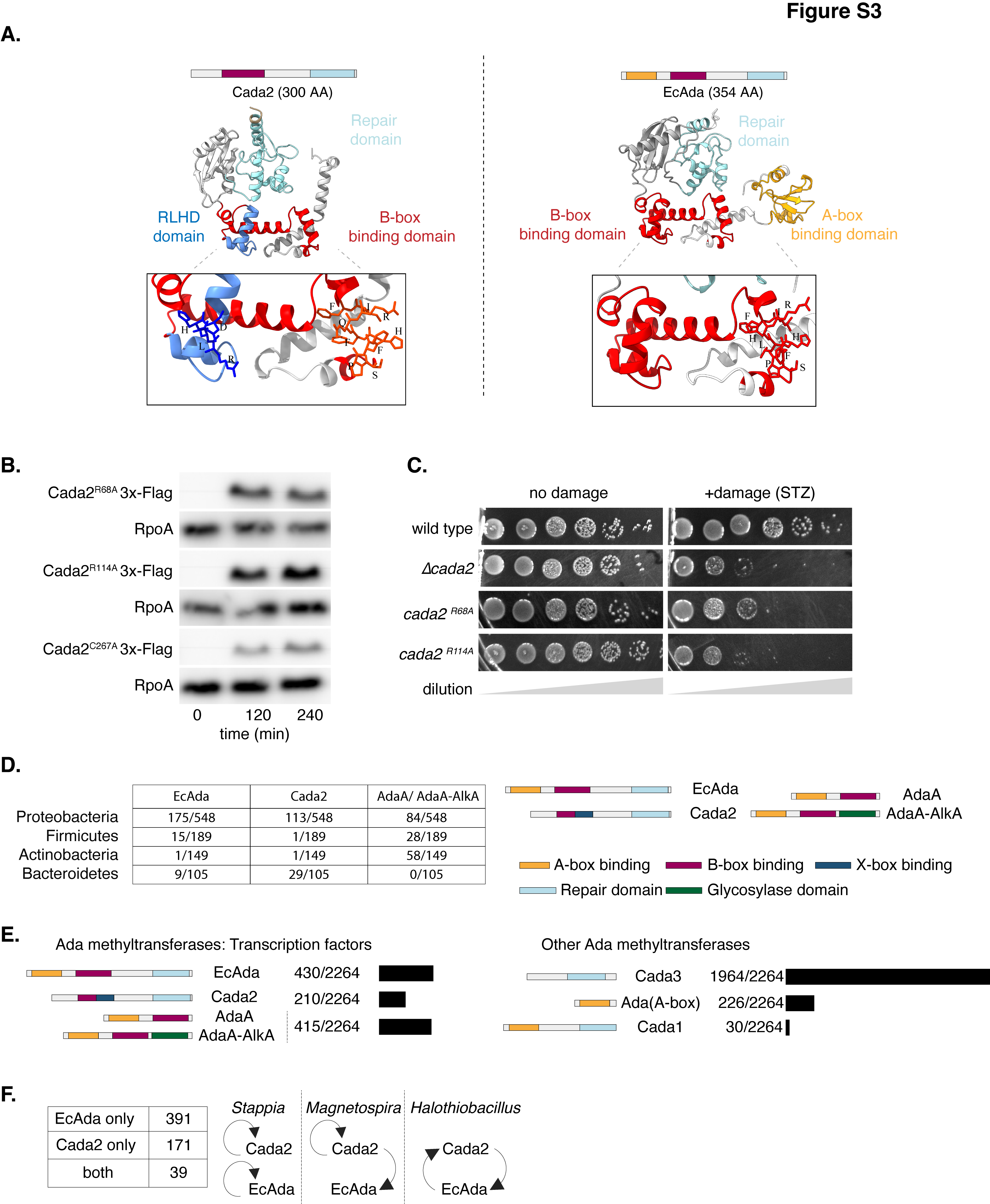

Supplement: S3 Fig — (A) (Top) Domain organization and predicted Alphafold structure of Cada2 and EcAda is indicated. (Bottom) Closeup of the EcAda and Cada2 regulatory domain reveals that the Cada2 B-box binding domain (possessing the “SPFHQR” amino acid sequence) and the newly identified sequence-specific binding domain of Cada2 (possessing the “RLHD” amino acid sequence) are part of a helix-turn-helix domain similar to the B-box binding domain of EcAda. The position of these conserved motifs are highlighted and labeled as a ball-and-stick in the overall ribbon representation of the models. (B) Western blot of flag-tagged Cada2 mutants (Cada2R68A and Cada2R114A) overexpressed in a Δcada2 strain from a xylose-inducible promoter treated with MMS damage. (C) Survival assay of cada2R68A and cada2R114A with or without STZ damage. (D) [Left] Prevalence of EcAda-like, Cada2-like, and AdaA-like proteins estimated from a curated, nonredundant database of bacterial genomes is analyzed at the genus level. Numbers represent the presence of these proteins for the major bacterial clades. [Right] domain organization of the respective adaptive response regulatory proteins analyzed here. (E) [Left] As (D) for a curated, nonredundant database of bacterial genomes at the species level. [Right] Prevalence of other adaptive response methyltransferases estimated from a curated, nonredundant database of bacterial genomes is analyzed at the species level. (F) Table represents presence and absence of EcAda-like and Cada2-like proteins and their co-occurrence. In the instances of Cada2-EcAda co-occurrence, potential regulatory circuits predicted via identifying Cada2 and EcAda binding motif in their cognate promoters are shown. (TIF) [file pbio.3002540.s003.tif]

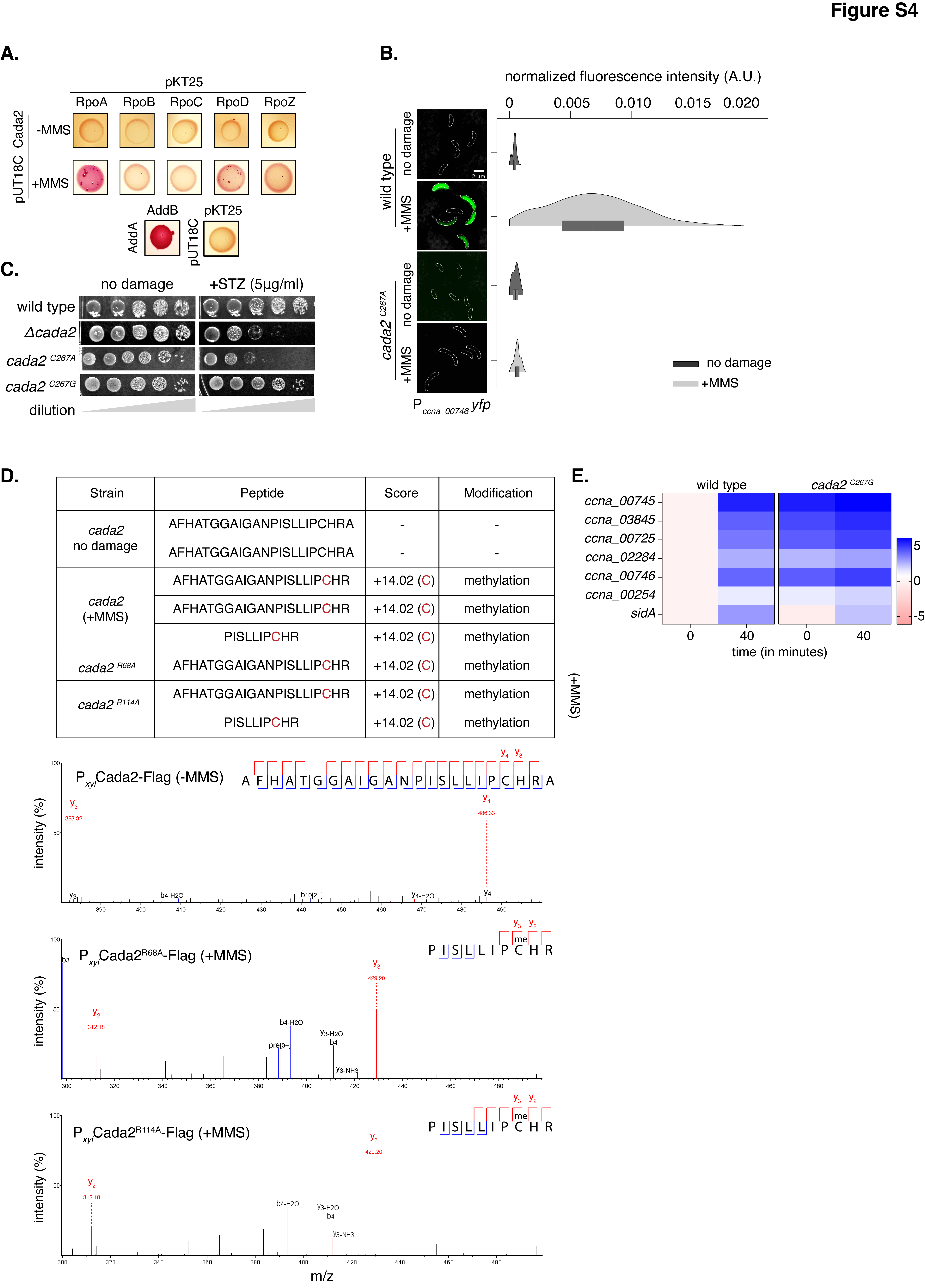

Supplement: S4 Fig — (A) Bacterial two-hybrid assay to test interaction between Cada2 and RNA polymerase subunits. T18-Cada2 was tested for interaction with T25-RNA polymerase holoenzyme subunits with and without 1.5 mM MMS. The presence of red colonies indicated positive interaction. As a positive control AddA (T18) and AddB (T25) are used, and empty vectors (T18 and T25) are used as negative control. Representative images from 2 independent repeats are shown. (B) [Left] Representative cells showing Pccna_00746-yfp reporter induction in wild type and cada2267A mutant background under 1.5 mM MMS damage. [Right] Violin plots showing fluorescence intensity distribution normalized to cell area from single cells in the presence (dark) and absence of damage (light) (n = 200, from 2 biological replicates). Wild-type data are represented again from Fig 1B. The underlying data are available in S1 Data. (C) Survival assay of the cada2 mutants (cada2C267A or cada2C267G) with and without STZ damage (5 μg/ml). (D) [Above] Table representing peptides corresponding to the Cada2 methyltransferase domain bearing the “PCHR” motif as identified via mass spectrometry. In the absence of MMS, peptides corresponding to wild-type Cada2-flag are unmethylated. Upon exposure to MMS damage, peptides corresponding to wild type as well as mutant Cada2-3x-Flag exhibit methylation at the Cys267 residue. [Below] Representative mass spectrometry fragmentation patterns for peptides in the above table. In case of wild type (no damage) representative spectrum from 3 fragments across 2 biological replicates is shown. No methylation modification on Cys267 was detected. In case of Cada2R68A representative spectrum from 4 fragments across 2 biological replicates is shown. Methylation modification on Cys267 was detected in 3 out of 4 fragments. In case of Cada2R114A representative spectrum from 7 fragments across 2 biological replicates is shown. Methylation modification on Cys267 was detected in 5 out of 7 fragments. (E) Heat [file pbio.3002540.s004.tif]

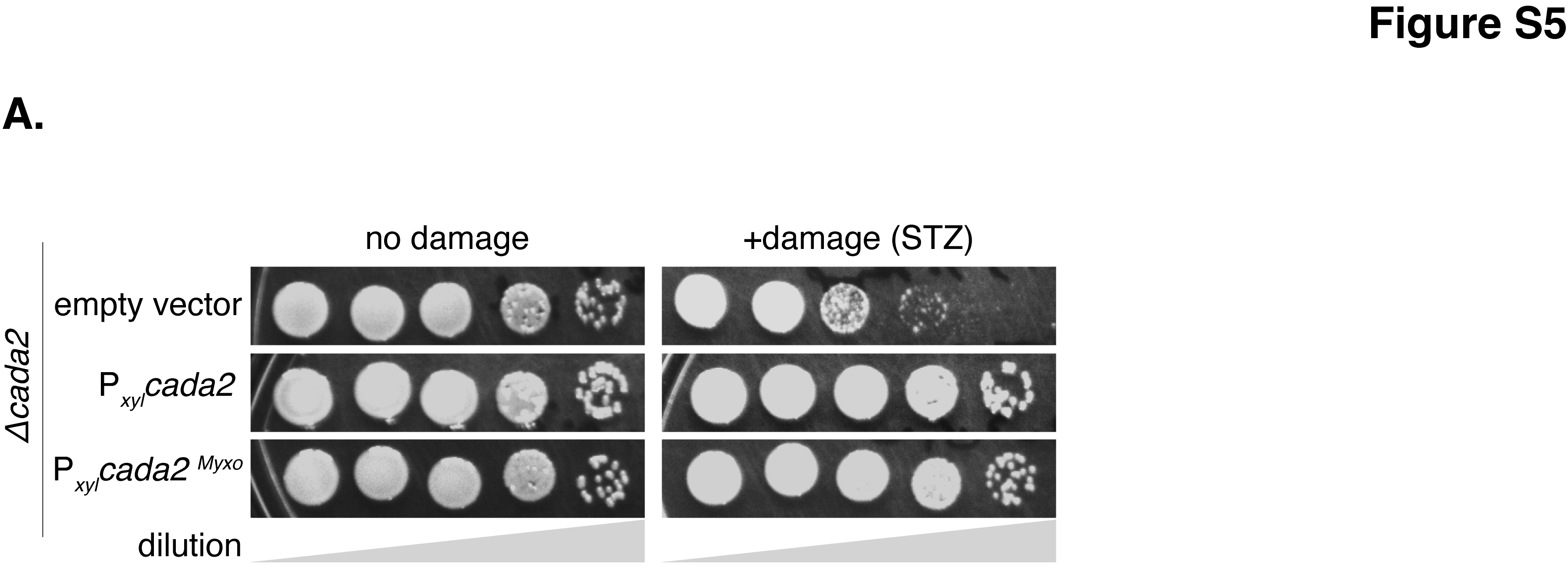

Supplement: S5 Fig — (A) Survival assay of Δcada2 strain overexpressing cada2caulo or cada2myxo (from xylose-inducible promoter) under methylation damage (5 μg/ml STZ). Survival of these strains was compared to a control strain comprising of an empty vector in a Δcada2 background. (TIF) [file pbio.3002540.s005.tif]
