# Supplementary material for: Widespread prevalence of a methylation-dependent switch to activate an essential DNA damage response in bacteria
Source: PLoS Biol. 2024 Mar 11;22(3):e3002540. doi: 10.1371/journal.pbio.3002540 (PMC10957082; doi:10.1371/journal.pbio.3002540)
Supplement: S1 Table — (DOCX) [file pbio.3002540.s006.docx]

**Table S1: Strains used in present study**

| **Strain** | **Genotype** | **Strain construction** |
| --- | --- | --- |
| CB15N | NA1000 | NA |
| NABC2 | *CB15N; ∆recA* | [1] |
| NABC581 | CB15N; P*_sidA_*-*YFP::kan^R^* | [2] |
| NABC477 | BTH101; pKT25-addB::kanR ; pUT18C-addA::carbR | [3] |
| NABC741 | CB15N; *rpoC-flag*::spec*^R^* | [4] |
| NABC735 | BTH101; pKT25; pUT18C | [5] |
| NABC815 | *CB15N; ∆driD* | [1] |
| NABC706 | CB15N; P*_ccna_00746_*-*YFP::kan^R^* | CB15N strain was transformed with pNABC735 plasmid to integrate *P_ccna_00746_-YFP* linked to *kan^R^* under the P*_xyl_* locus. |
| NABC707 | *CB15N; ∆cada1* | CB15N was transformed with pNABC736 plasmid to generate deletion of *cada1* through two-step recombination procedure (Skerker et al., 2005). |
| NABC708 | *CB15N; ∆cada2* | CB15N was transformed with pNABC737 plasmid to generate deletion of *cada2* through two-step recombination procedure (Skerker et al., 2005). |
| NABC709 | *CB15N; ∆cada3* | CB15N was transformed with pNABC738 plasmid to generate deletion of *cada3* through two-step recombination procedure (Skerker et al., 2005). |
| NABC710 | *CB15N; ∆cada1;* P*_ccna_00746_*-*YFP::kan^R^* | NABC707 strain was transformed with pNABC735 plasmid to integrate P*_ccna_00746_*-*YFP* at the *xyl* locus. |
| NABC711 | *CB15N; ∆cada2;* P*_ccna_00746_*-*YFP::kan^R^* | NABC708 strain was transformed with pNABC735 plasmid to integrate P*_ccna_00746_*-*YFP* at the *xyl* locus. |
| NABC712 | *CB15N; ∆cada3;* P*_ccna_00746_*-*YFP::kan^R^* | NABC709 strain was transformed with pNABC735 plasmid to integrate P*_ccna_00746_*-*YFP* at the *xyl* locus. |
| NABC713 | *CB15N; ∆cada2;* pMT687-P*_xyl_-cada2::gent^R^* | NABC708 was transformed with pNABC739 plasmid. |
| NABC714 | CB15N; *cada2 (C267A)* | CB15N was transformed with *pNABC740* plasmid to generate a specific mutation in the conserved C267 residue of *cada2* through two-step recombination procedure (Skerker et al., 2005). |
| NABC715 | CB15N; *cada2 (C267A); P_ccna_00746_-YFP::kan^R^* | NABC714 strain was transformed with pNABC735 plasmid to integrate P*_ccna_00746_*-*YFP* at the *xyl* locus. |
| NABC716 | CB15N; *cada2 (C267G)* | CB15N was transformed with p*NABC741* plasmid to generate deletion of *cada1* through two-step recombination procedure (Skerker et al., 2005). |
| NABC717 | CB15N; *cada2 (C267G);* P*_ccna_00746_-YFP::kan^R^* | NABC716 strain was transformed with pNABC735 plasmid to integrate Pccna_00746-YFP at the *xyl* locus. |
| NABC718 | CB15N; *cada2-flag*::spec*^R^* | CB15N strain was transformed with pNABC742 plasmid to integrate *cada2-flag* linked to *spec^R^* under the endogenous *cada2* locus. |
| NABC719 | CB15N; P*_ccna_00746_*-*YFP(scramble)::kan^R^* | CB15N strain was transformed with pNABC743 plasmid to integrate *P_ccna_00746_-YFP(scramble)* linked to *kan^R^* under the P*_xyl_* locus. |
| NABC720 | CB15N; P*_ccna_00746_*-*YFP(AT-rich)::kan^R^* | CB15N strain was transformed with pNABC744 plasmid to integrate P*_ccna_00746_*-*YFP(AT-rich)* linked to *kan^R^* under the P*_xyl_* locus. |
| NABC721 | CB15N; *cada2 (R68A)* | CB15N was transformed with *cada2 pNABC745* plasmid to generate a specific mutation in the conserved R68 residue of *cada2* through two-step recombination procedure (Skerker et al., 2005). |
| NABC722 | CB15N; *cada2 (R68A); P_ccna_00746_-YFP::kan^R^* | NABC721 strain was transformed with pNABC735 plasmid to integrate P*_ccna_00746_*-YFP at the *xyl* locus. |
| NABC723 | CB15N; *cada2 (R114A)* | CB15N was transformed with pNABC746 plasmid to generate a specific mutation in the conserved R114 residue of *cada2* through two-step recombination procedure (Skerker et al., 2005). |
| NABC724 | CB15N; *cada2 (R114A); P_ccna_00746_-YFP::kan^R^* | NABC723 strain was transformed with pNABC735 plasmid to integrate P*_ccna_00746_*-*YFP* at the *xyl* locus. |
| NABC725 | *CB15N; ∆cada2;*pMT463-P*_xyl_-cada2-flag::gent^R^* | NABC708 was transformed with pNABC747 plasmid. |
| NABC726 | *CB15N; ∆cada2;*pMT463-P*_xyl_-cada2(R68A)-flag::gent^R^* | NABC708 was transformed with pNABC748 plasmid. |
| NABC727 | *CB15N; ∆cada2;*pMT687-P*_xyl_-cada2(R114A)-flag::gent^R^* | NABC708 was transformed with pNABC749 plasmid. |
| NABC728 | *CB15N; ∆cada2;*pMT687-P*_xyl_-cada2(C267A)-flag::gent^R^* | NABC708 was transformed with pNABC750 plasmid. |
| NABC729 | *CB15N; ∆cada2;*pMT687-*P_xyl_-cada2^myxococcus^::gent^R^* | NABC708 was transformed with pMT463-pNABC751 plasmid. |
| NABC730 | *CB15N; ∆cada2;*pMT687-P*_xyl_-cada2^myxococcus^::gent^R^;* P*_ccna_00746_-YFP::kan^R^* | NABC711 was transformed with pNABC751 plasmid. |
| NABC731 | *CB15N; ∆cada2;*pMT687-*P_xyl_-EcAda::gent^R^;* P*_ccna_00746_-YFP::kan^R^* | NABC711 was transformed with pNABC752 plasmid. |
| NABC732 | *CB15N; ∆cada2;*P*_EcAda_-YFP::kan^R^* | NABC708 strain was transformed with pNABC753 plasmid to integrate P*_EcAda_*-*YFP* at the *xyl* locus. |
| NABC733 | *CB15N; ∆cada2;*pMT687-P*_xyl_-cada2^myxococcus^::gentR;* P*_EcAda_-YFP::kan^R^* | NABC732 was transformed with pNABC751 plasmid. |
| NABC734 | *CB15N; ∆cada2;*pMT687-P*_xyl_-EcAda::gent^R^;* P*_EcAda_-YFP::kan^R^* | NABC732 was transformed with pNABC752 plasmid. |
| NABC736 | BTH101; pKT25-rpoA::kan^R^ ; pUT18C-cada2::carb^R^ | BTH101 was co-transformed with pNABC754 and pNABC755 for the bacterial-two-hybrid assay |
| NABC737 | BTH101; pKT25-rpoB::kan^R^ ; pUT18C-cada2::carb^R^ | BTH101 was co-transformed with pNABC754 and pNABC756 for the bacterial-two-hybrid assay |
| NABC738 | BTH101; pKT25-rpoC::kan^R^ ; pUT18C-cada2::carb^R^ | BTH101 was co-transformed with pNABC754 and pNABC757 for the bacterial-two-hybrid assay |
| NABC739 | BTH101; pKT25-rpoD::kan^R^ ; pUT18C-cada2::carb^R^ | BTH101 was co-transformed with pNABC754 and pNABC758 for the bacterial-two-hybrid assay |
| NABC740 | BTH101; pKT25-rpoZ::kan^R^ ; pUT18C-cada2::carb^R^ | BTH101 was co-transformed with pNABC754 and pNABC759for the bacterial-two-hybrid assay |
| NABC819 | CB15N; ∆driD; P_ccna_00746_-YFP::kan^R^ | NABC815 strain was transformed with pNABC735 plasmid to integrate P*_ccna_00746_*-*YFP* at the *xyl* locus. |

**References:**

1. Modell JW, Kambara TK, Perchuk BS, Laub MT: **A DNA Damage-Induced, SOS-Independent Checkpoint Regulates Cell Division in *Caulobacter crescentus***. *PLoS Biology* 2014, **12**:e1001977.

2. Joseph AM, Nahar K, Daw S, Hasan MM, Lo R, Le TBK, Rahman KM, Badrinarayanan A: **Mechanistic insight into the repair of C8-linked pyrrolobenzodiazepine monomer-mediated DNA damage**. *RSC Med Chem* 2022, doi:10.1039/D2MD00194B.

3. Badrinarayanan A, Le TBK, Spille J-H, Cisse II, Laub MT: **Global analysis of double-strand break processing reveals in vivo properties of the helicase-nuclease complex AddAB**. *PLOS Genetics* 2017, **13**:e1006783.

4. Haakonsen DL, Yuan AH, Laub MT: **The bacterial cell cycle regulator GcrA is a σ70 cofactor that drives gene expression from a subset of methylated promoters**. *Genes Dev* 2015, **29**:2272–2286.

5. Chimthanawala A, Parmar JJ, Kumar S, Iyer KS, Rao M, Badrinarayanan A: **SMC protein RecN drives RecA filament translocation for in vivo homology search**. *Proc Natl Acad Sci U S A* 2022, **119**:e2209304119.
