# Supplementary material for: Widespread prevalence of a methylation-dependent switch to activate an essential DNA damage response in bacteria
Source: PLoS Biol. 2024 Mar 11;22(3):e3002540. doi: 10.1371/journal.pbio.3002540 (PMC10957082; doi:10.1371/journal.pbio.3002540)
Supplement: S2 Table — (DOCX) [file pbio.3002540.s007.docx]

**Table S2: Plasmids used in present study**

| **Plasmid** | **Construction details** | **Antibiotic** |
| --- | --- | --- |
| pNPTS138 | [1] | Kanamycin |
| pXYFPC2 | [2] | Kanamycin |
| pBXMCS4 | [2] | Gentamicin |
| pMCS1 | [2] | Spectinomycin |
| pNABC735 | pXYFPC2 vector was amplified using AB_oligo_651 and AB_oligo_171 and P*_ccna_00746_*fragment was amplified from *Caulobacter* genomic DNA (gDNA). The vector and insert fragments were assembled with Gibson assembly. | Kanamycin |
| pNABC736 | 600 bp fragments upstream and downstream of *cada1*(*ccna_00746*) genomic locus were amplified from *C. crescentus* gDNA. Primers pairs used for amplification were AMJ_oligo_047/AMJ_oligo_048 (upstream fragment) and AMJ_oligo_049/AMJ_oligo_050 (downstream fragment). These fragments were assembled with BamH1/Nhe1 linearized pNPTS138 vector using Gibson assembly. | Kanamycin |
| pNABC737 | 600 bp fragments upstream and downstream of *cada1*(*ccna_03845*) genomic locus were amplified from *C. crescentus* gDNA. Primers pairs used for amplification were AK_oligo_84/AK_oligo_85 (upstream fragment) and AK_oligo_86/AK_oligo_87 (downstream fragment). These fragments were assembled with BamH1/Nhe1 linearized pNPTS138 vector using Gibson assembly. | Kanamycin |
| pNABC738 | 600 bp fragments upstream and downstream of *cada1*(*ccna_00725*) genomic locus were amplified from *C. crescentus* gDNA. Primers pairs used for amplification were AK_oligo_174/AK_oligo_176(upstream fragment) and AK_oligo_175/AK_oligo_177 (downstream fragment). These fragments were assembled with BamH1/Nhe1 linearized pNPTS138 vector using Gibson assembly. | Kanamycin |
| pNABC739 | Full length *cada2* was amplified from the *C. crescentus* gDNA using AK_oligo_114 and AK_oligo_247. The amplified fragment and pBXMCS4 were both digested with NdeI/EcoRI and ligated to assemble the required construct. | Gentamicin |
| pNABC740 | 600bp upstream of the mutation site (C267) of *cada2* was amplified using AK_oligo_182 and AK_oligo_169 from *C. crescentus* gDNA. 600bp downstream of the mutation site (C267) of *cada2* was amplified using AK_oligo_183 and AK_oligo_168 from *C. crescentus* gDNA. pNPTS138 vector was digested with BamHI/NheI. The upstream fragment, downstream fragment and digested vector were assembled using Gibson assembly. AK_oligo_168 and AK_oligo_169 are overlapping oligos which harbor the desired mutant sequence. | Kanamycin |
| pNABC741 | 600bp upstream of the mutation site (C267) of *cada2* was amplified using AK_oligo_182 and AK_oligo_169 from *C. crescentus* gDNA. 600bp downstream of the mutation site (C267) of *cada2* was amplified using AK_oligo_183 and AK_oligo_168 from *C. crescentus* gDNA. pNPTS138 vector was digested with BamHI/NheI. The upstream fragment, downstream fragment and digested vector were assembled using Gibson assembly. AK_oligo_168 and AK_oligo_169 are overlapping oligos which harbor the desired mutant sequence. | Kanamycin |
| pNABC742 | Full length *cada2* was amplified from the *C. crescentus* gDNA using AK_oligo_114 and AK_oligo_115. The amplified fragment and pMCS1 were both digested with NdeI/EcoRI and ligated to assemble the required construct. | Spectinomycin |
| pNABC743 | pXYFPC2 vector was amplified using AB_oligo_651 and AB_oligo_652. P*_ccna_00746_*-*yfp* (scramble) was amplified from pNABC735 by using overlapping primer pairs with the scrambled sequnce (AK_oligo_49,AK_oligo_260 and AK_oligo_259, AC_oligo_321). | Kanamycin |
| pNABC744 | pXYFPC2 vector was amplified using AB_oligo_651 and AB_oligo_652. P*_ccna_00746_*-*yfp* (AT-rich) was amplified from pNABC735 by using overlapping primer pairs with the scrambled sequnce (AK_oligo_49,AK_oligo_258 and AK_oligo_257, AC_oligo_321). | Kanamycin |
| pNABC745 | 600bp upstream of the mutation site (R68) of *cada2* was amplified using AK_oligo_220 and AK_oligo_221 from *C. crescentus* gDNA. 600bp downstream of the mutation site (R68) of *cada2* was amplified using AK_oligo_222 and AK_oligo_223 from *C. crescentus* gDNA. pNPTS138 vector was digested with BamHI/NheI. The upstream fragment, downstream fragment and digested vector were assembled using Gibson assembly. AK_oligo_221 and AK_oligo_222 are overlapping oligos which harbor the desired mutant sequence. | Kanamycin |
| pNABC746 | 600bp upstream of the mutation site (R114) of *cada2* was amplified using AK_oligo_252 and AK_oligo_255 from *C. crescentus* gDNA. 600bp downstream of the mutation site (R114) of *cada2* was amplified using AK_oligo_253 and AK_oligo_254 from *C. crescentus* gDNA. pNPTS138 vector was digested with BamHI/NheI. The upstream fragment, downstream fragment and digested vector were assembled using Gibson assembly. AK_oligo_254 and AK_oligo_255 are overlapping oligos which harbor the desired mutant sequence. | Kanamycin |
| pNABC747 | *pBXMCS4 vector was amplified using KM_oligo_72 and KM_oligo_73. cada2 flag* fragment was amplified from *cada2 flag* strain using AK_oligo_278 and AK_oligo_279. The vector and insert fragments were assembled with Gibson assembly. | Gentamicin |
| pNABC748 | NABC747 vector was amplified using AK_oligo_73 and AK_oligo_284. Full length *cada2^R68A^* was amplified from the *NABC721* gDNA using AK_oligo_278 and AK_oligo_283. | Gentamicin |
| pNABC749 | NABC747 vector was amplified using AK_oligo_73 and AK_oligo_284. Full length *cada2^R114A^* was amplified from the *NABC721* gDNA using AK_oligo_278 and AK_oligo_283. | Gentamicin |
| pNABC750 | NABC747 vector was amplified using AK_oligo_73 and AK_oligo_284. Full length *cada2^R68A^* was amplified from the *NABC714* gDNA using AK_oligo_278 and AK_oligo_283. | Gentamicin |
| pNABC751 | Full length *cada2^myxo^* was amplified from the *myxococcus xanthusDK1622* gDNA using AK_oligo_263 and AK_oligo_264. The amplified fragment and pBXMCS4 were both digested with NdeI/EcoRI and ligated to assemble the required construct. | Gentamicin |
| pNABC752 | pBXMCS4 vector was amplified using KM_oligo_73 and AK_oligo_280. Full length *EcAda* was amplified from the *E.coli* gDNA using AK_oligo_281 and AK_oligo_282. The vector and insert fragments were assembled with Gibson assembly. | Gentamicin |
| pNABC753 | pXYFPC2 vector was amplified using AB_oligo_651 and AB_oligo_171 and P*_EcAda_* fragment was amplified from *Caulobacter* genomic DNA (gDNA) using AK_oligo_264 and AK_oligo_265. The vector and insert fragments were assembled with Gibson assembly. | Kanamycin |
| pNABC754 | Full length *cada2* was amplified from the *C. crescentus* gDNA using AK_oligo_94 and AK_oligo_108. The amplified fragment and pUT18C were both digested with BamHI/KpnI and ligated to assemble the required construct. | Carbenicillin |
| pNABC755 | Full length *rpoA* was amplified from the *C. crescentus* gDNA using AC_oligo_112 and AK_oligo_113. The amplified fragment and pkT25 were both digested with BamHI/KpnI and ligated to assemble the required construct. | Kanamycin |
| pNABC756 | Full length *rpoB* was amplified from the *C. crescentus* gDNA using AC_oligo_114 and AK_oligo_115. The amplified fragment and pkT25 were both digested with BamHI/KpnI and ligated to assemble the required construct. | Kanamycin |
| pNABC757 | Full length *rpoC* was amplified from the *C. crescentus* gDNA using AC_oligo_116 and AK_oligo_117. The amplified fragment and pkT25 were both digested with BamHI/KpnI and ligated to assemble the required construct. | Kanamycin |
| pNABC758 | Full length *rpoD* was amplified from the *C. crescentus* gDNA using AC_oligo_118 and AK_oligo_119. The amplified fragment and pkT25 were both digested with BamHI/KpnI and ligated to assemble the required construct. | Kanamycin |

**References:**

1. Skerker JM, Laub MT: **Cell-cycle progression and the generation of asymmetry in *Caulobacter crescentus***. *Nat Rev Microbiol* 2004, **2**:325–337.

2. Thanbichler M, Iniesta AA, Shapiro L: **A comprehensive set of plasmids for vanillate- and xylose-inducible gene expression in *Caulobacter crescentus***. *Nucleic Acids Res* 2007, **35**:e137.
