# Supplementary material for: Widespread prevalence of a methylation-dependent switch to activate an essential DNA damage response in bacteria
Source: PLoS Biol. 2024 Mar 11;22(3):e3002540. doi: 10.1371/journal.pbio.3002540 (PMC10957082; doi:10.1371/journal.pbio.3002540)
Supplement: S3 Table — (DOCX) [file pbio.3002540.s008.docx]

**Table S3: Oligos used in present study**

| **Oligo** | **Sequence** |
| --- | --- |
| AK_oligo_49 | GGTCAGGTCGGTCATGGGCAAGAGGTCCAGTTCGGCGAGCCGCGCGCCAT |
| AK_oligo_140 | CTCCTCGCCCTTGCTCACCATCGGAAGGTCTCCTTTCGTGAGGAGACCATG |
| AB_oligo_651 | CTGGACCTCTTGCCCATGACCGA |
| AB_oligo_566 | ATGGTGAGCAAGGGCGAGGAG |
| AK_oligo_182 | CAAGCTTCTCTGCAGGATATCTGCGGAACTGCAGGGCCGCCTG |
| AK_oligo_183 | CGGAGACGCGTCACGGCCGAAGCAGCGCATCTTCACCCGCTGG |
| AK_oligo_168 | CGCTGCTGATCCCCGCCCACCGCGCCATCGCCAAGG |
| AK_oligo_169 | GCGATGGCGCGGTGGGCGGGGATCAGCAGCGAGATGGG |
| AMJ_oligo_047 | CAAGCTTCTCTGCAGGATATCTGGTTCATGCAGGGCAACAAGGACCTGGC |
| AMJ_oligo_048 | GTCAGACCCCGTAGAAAAGATCGTTCCAGCGGGCGTCGTCGTC |
| AMJ_oligo_049 | GACCCAAGTACCGCCACCTAAGTGGCGCGCAAGCGTGCGTTG |
| AMJ_oligo_050 | CGGAGACGCGTCACGGCCGAAGCGTCGGCCGGGCGCTCCC |
| AK_oligo_84 | CAAGCTTCTCTGCAGGATATCTG GGAGTCTCGACCATGGGGCGACCAT |
| AK_oligo_85 | CTAGGGGGAGGATCAGGAAGCCGCCGCGACGTCATAAGCCATGGTCATAGGCTAGGCCCC |
| AK_oligo_86 | GTCGCGGCGGCTTCCTGATCCTCC |
| AK_oligo_87 | CGGAGACGCGTCACGGCCGAAG AGGGCACGACGGTCTCGACCAA |
| AK_oligo_174 | CAAGCTTCTCTGCAGGATATCTGCGCCCTGCTGCTGGGCGAAGGC |
| AK_oligo_175 | CGGAGACGCGTCACGGCCGAAGGGCCGTCATCGCCTGCGTCA |
| AK_oligo_176 | ACTCTAGAGCGCCCCTTCGTGGGCCAGCAGAAAGGGGCCGCCACAGCGAC |
| AK_oligo_177 | CTGCTGGCCCACGAAGGGGC |
| AK_oligo_114 | TTATCATATGACCATGGCTTATGACGCATCTTCCCTTTCC |
| AK_oligo_115 | TTATGAATTCTCGGAAGCCGCCGCGACCGCCT |
| AK_oligo_247 | TTATGAATTCTCAGGAAGCCGCCGCGACC |
| AK_oligo_259 | CACGTCCGGGTAGCGGGCGAACGCGTGTCGAATGATCACGGACGCGGCCT |
| AK_oligo_260 | CGACACGCGTTCGCCCGCTACCCGGACGTGTC |
| AK_oligo_257 | CCGCAAGGTTAATAATGATCACGGACGCGGCCTAGCG |
| AK_oligo_258 | CGTGATCATTATTAACCTTGCGGCCCGCTACCCGGACG |
| AK_oligo_220 | CAAGCTTCTCTGCAGGATATCTGTGATCCGCGCTCCGCCGG |
| AK_oligo_221 | GTGAAGATGGCCTGGAAATGGAACGGCGAAAGGCC |
| AK_oligo_222 | TTCCATTTCCAGGCCATCTTCACCCGCTGGGCGGG |
| AK_oligo_223 | CGGAGACGCGTCACGGCCGAAGGTGACAGGGGATCAGCAGCGAG |
| AK_oligo_252 | CAAGCTTCTCTGCAGGATATCTGAAGACGAGAAGGGCGCGCGC |
| AK_oligo_253 | CGGAGACGCGTCACGGCCGAAGCCGGTCGCTTCGCGACCACC |
| AK_oligo_254 | CCGGGCTTTCGGGACCCTCGGCCCTGCATGACCTGTTCATCGCTCAGG |
| AK_oligo_255 | CATGCAGGGCCGAGGGTCCCGAAAGCCCGG |
| AK_oligo_263 | TTATCATATGTTGCTCCACCATGTGTTGGCGGA |
| AK_oligo_264 | TTATGAATTCTCACATACCGCTGAGCGGCG |
| AK_oligo_281 | ACTAGTGGATCCCCCGGGCTGCAGGAATTCTTACCTCTCCTCATTTTCAGCTTCGCGG |
| AK_oligo_282 | CAGACGCTCGAGTTTTGGGGAGACGACCATATGAAAAAAGCCACATGCTTAACTGACGATC |
| AK_oligo_274 | GGTCAGGTCGGTCATGGGCAAGAGGTCCAGGGAGAAAGCTAAAGAGGTTGTTCGCCG |
| AK_oligo_275 | GGTGAACAGCTCCTCGCCCTTGCTCACCATAATCAGCTCCCTGGTTAAGGATAGCCT |
| AK_oligo_278 | ACGCTCGAGTTTTGGGGAGACGACCATATGACCATGGCTTATGACGCATCTTCCC |
| AK_oligo_283 | GGAAGCCGCCGCGACCGC |
| AK_oligo_284 | CTGGGCATGGAGGCGGTCGCGGCGGCTTCCGAGAATTCGAACGTTACGCGTCACCG |
| AK_oligo_94 | TTATGGATCCC ATGACCATGGCTTATGACGCATCTTCCCTTT |
| AK_oligo_108 | TTATGGTACCCGGGAAGCCGCCGCGACCGCC |
| AC_oligo112 | TTATGGATCCCGTGATCGAAAGAAACTGGAACGAGCTGATCCGTCCTGAGAAGCCGC |
| AC_oligo113 | TTATGGTACCTTAGATCTGGTCTTCGAACTTCTTGGCCAGGTCTTCGATGTTCTCCGGCG |
| AC_oligo114 | TTATGGATCCCATGGCGCAATCCTTCACCGGCAAGAAGCGG |
| AC_oligo115 | TTATGGTACCTCAGCTGTTCTCCAGCTCGACGTTCAGGCCGAGCG |
| AC_oligo116 | TTATGGATCCCATGAACCAGGAAGTCCTGAACATCTTCAATCCGGTCCAGGCCG |
| AC_oligo117 | TTATGGTACCCTATTCGGCGTCCGAAAGCGCGATCTCGGCC |
| AC_oligo118 | TTATGGATCCCATGAGCAACAATTCCTCGGCCGAGACGGAAGCGC |
| AC_oligo119 | TTATGGTACCTTACGAGTCCAGGAAGCTGCGCAGCTTGCGCGAC |
