# Supplementary material for: Widespread prevalence of a methylation-dependent switch to activate an essential DNA damage response in bacteria
Source: PLoS Biol. 2024 Mar 11;22(3):e3002540. doi: 10.1371/journal.pbio.3002540 (PMC10957082; doi:10.1371/journal.pbio.3002540)
Supplement: S4 Table — (DOCX) [file pbio.3002540.s009.docx]

**Table S4: Kits used for preparing RNA libraries and sequencing platform used for the RNA-seq experiment**

| **Background** | **treatment condition** | **RNA library preparation kit** | **Sequencing platform** |
| --- | --- | --- | --- |
| *wild type* | MMS | NEBNext® Ultra™ II Directional RNA Library Prep with Sample Purification Beads (Catalog no-E7765L) | Illumina Hiseq2500 |
| *ΔrecA* | MMS |  |  |
| *Δcada1* | MMS |  |  |
| *Δcada2* | MMS |  |  |
| *wild type* | MMC | NEBNext® Ultra™ II Directional RNA Library Prep with Sample Purification Beads (Catalog no-E7765L) | Illumin Novaseq4000 |
| *wild type* | Norfloxacin |  |  |
