# Supplementary material for: Widespread prevalence of a methylation-dependent switch to activate an essential DNA damage response in bacteria
Source: PLoS Biol. 2024 Mar 11;22(3):e3002540. doi: 10.1371/journal.pbio.3002540 (PMC10957082; doi:10.1371/journal.pbio.3002540)
Supplement: S1 Raw Images — (PDF) [file pbio.3002540.s011.pdf]

Figure S2.B Anti-flag

Without contrast_raw

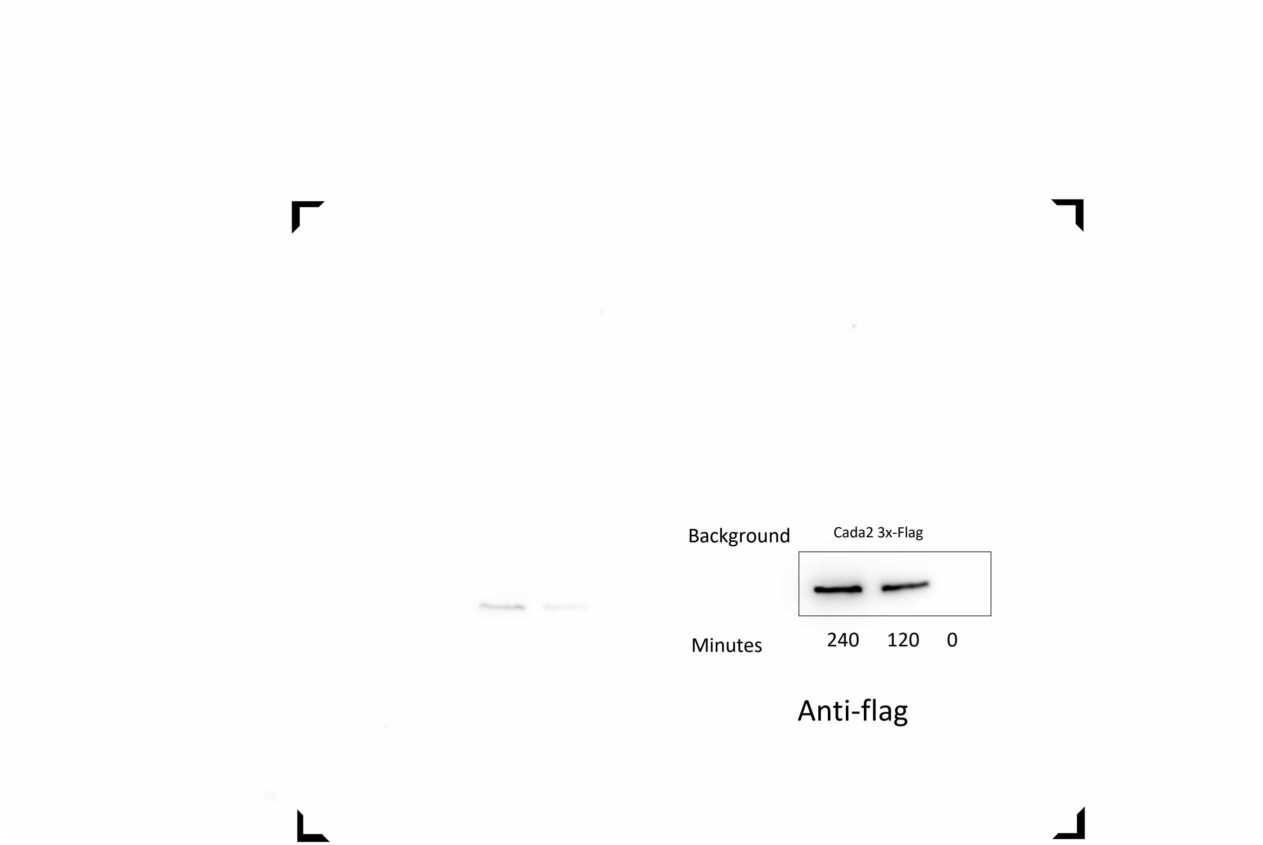

With contrast and ladder overlaid

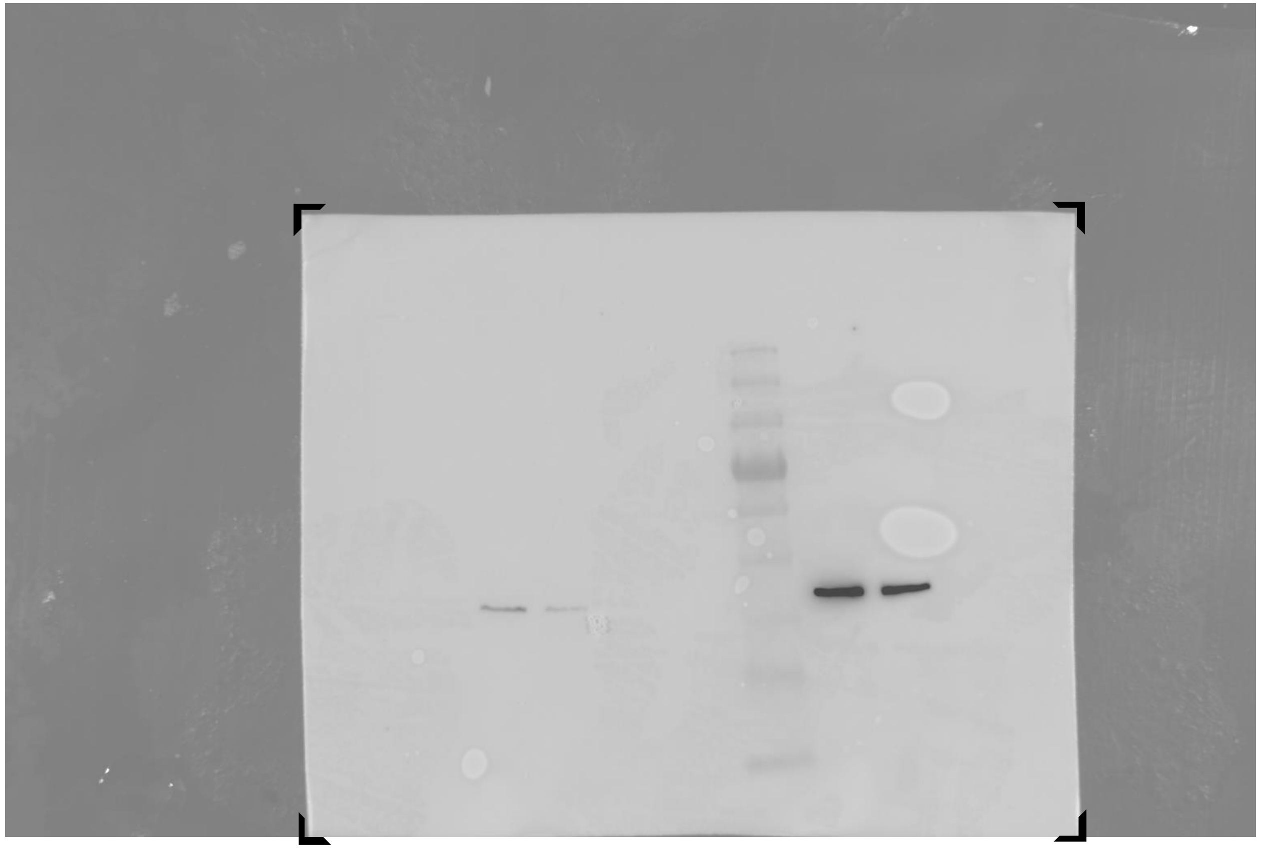

Figure S2.B Anti-RpoA

Without contrast_raw

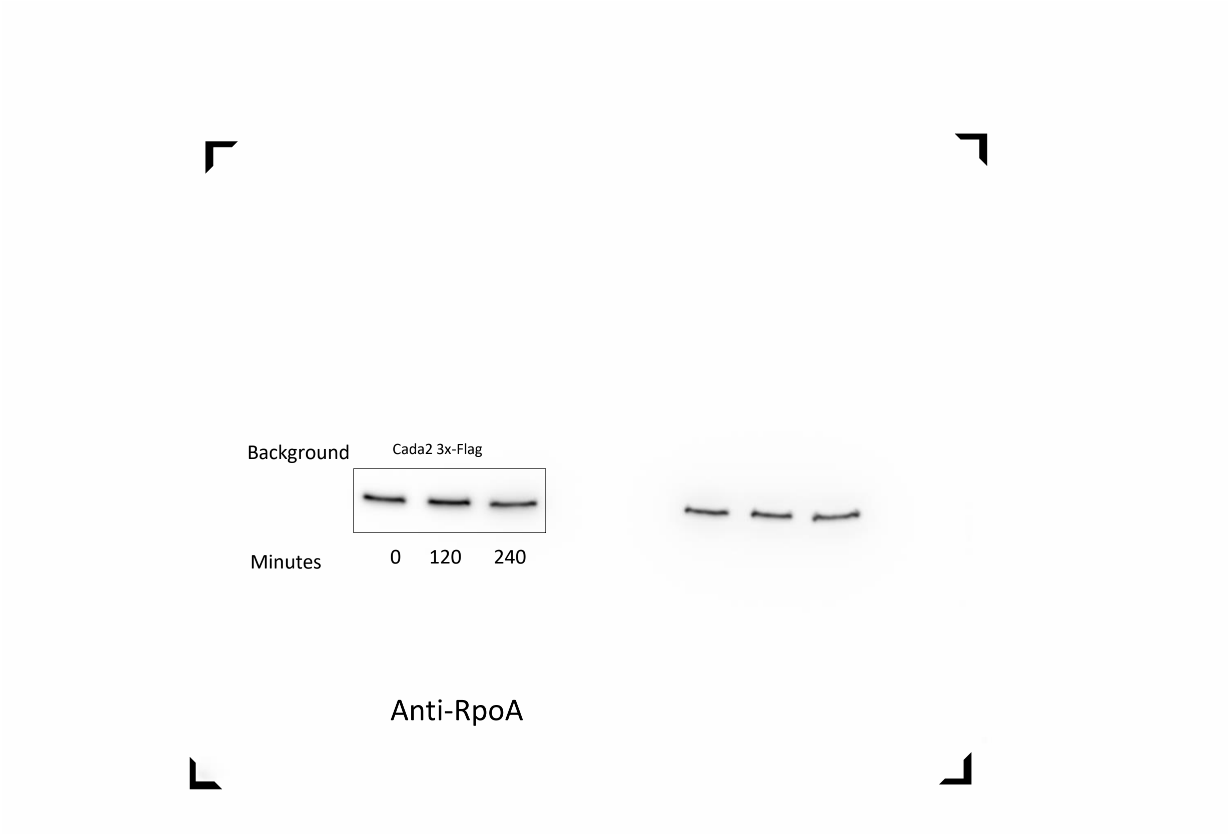

With contrast and ladder overlaid

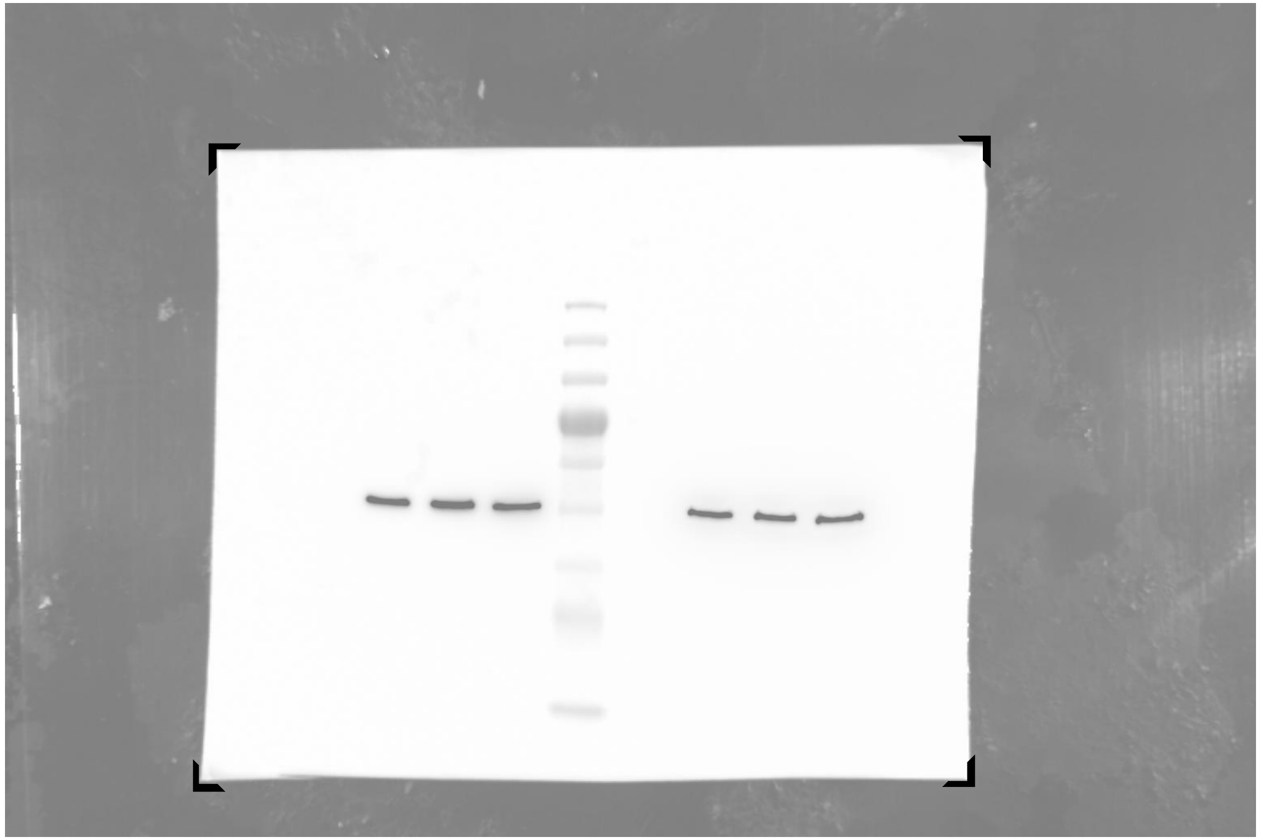

Figure S3.B

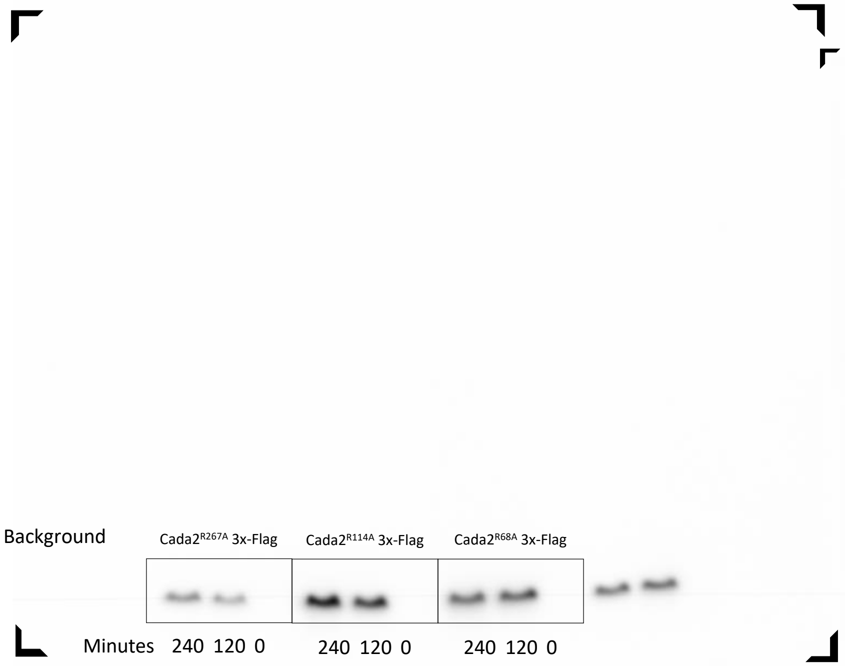

Anti-flag

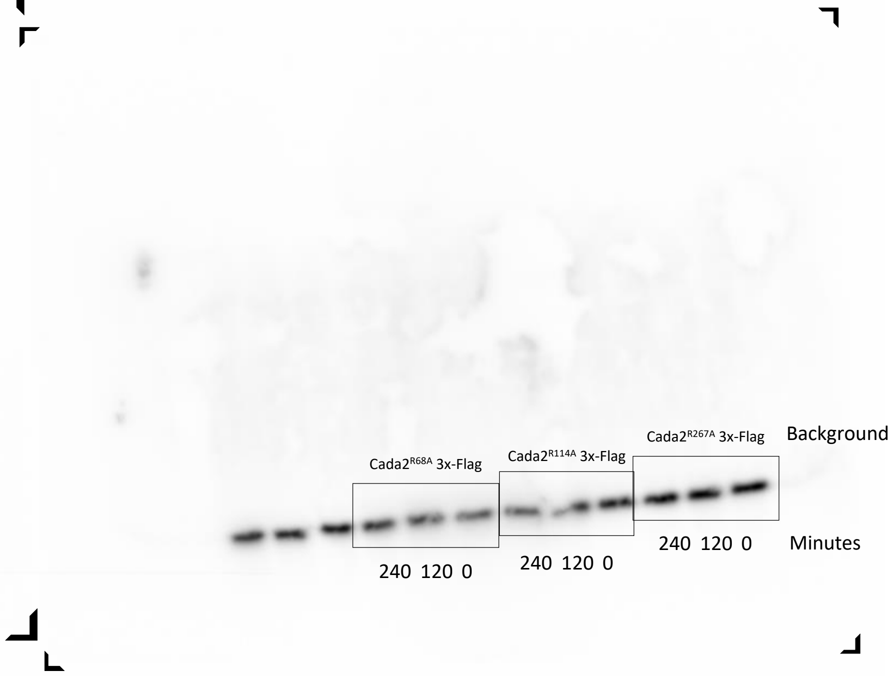

Anti-RpoA

Without contrast_raw

Figure S3.B

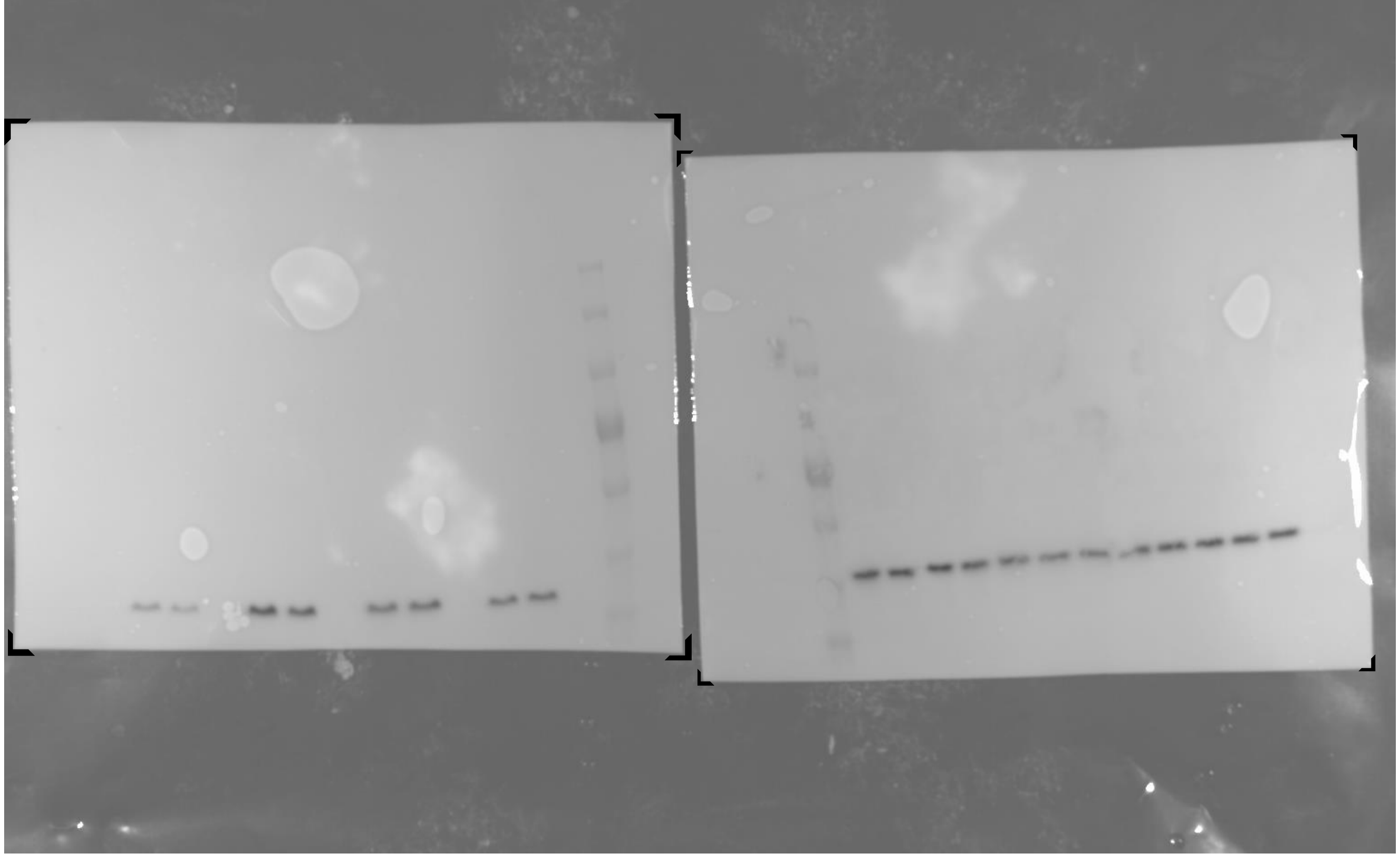

With contrast and ladder overlaid
